# Bioeconomic efficiency of pasture-based production systems in the dairy cattle chain of the Pre-Amazon region of Maranhão

**DOI:** 10.1007/s11250-026-05060-3

**Published:** 2026-05-08

**Authors:** Allan Stênio da Silva Santos, Marcos Jácome Araújo, Clésio dos Santos Costa, Francisco Naysson de Sousa Santos, Henrique Nunes Parente, Marcônio Martins Rodrigues, Hêmylle Jhec Santos Menêses, Maria Dulceyelena Calixto de Sousa, Débora Cristina Furtado da Silva, Aline Nascimento Ferreira

**Affiliations:** 1 Agriculture and Environmental Science Center, Federal University of Piauí, BR 135, Km 3, Professora Cinobelina Elvas Campus, Bom Jesus, Piauí, 64.900-000 Brazil; 2 Animal Science Department, Federal University of Ceara, 2977 Mister Hull Avenue, Pici Campus - Blocks 808-810, Fortaleza, Ceara 60.356-000 Brazil; 3 Agriculture and Environmental Sciences Center, Federal University of Maranhão, BR 222, Km 4, University Campus, Chapadinha, Maranhão 65.500-000 Brazil; 4 Animal Science Department, Federal University of Piauí (UFPI), Ministro Petrônio Portella Campus, Ininga, Teresina/PI 64049-550 Brazil

**Keywords:** Economic analysis, Rural management, Livestock systems, Sustainability

## Abstract

The search for greater profitability and sustainability in dairy cattle farming drives the adoption of different production strategies, especially on small farms. The objective of this study was to evaluate the bioeconomic efficiency of pasture-based dairy production systems on farms of up to 70 hectares in the Pre-Amazon region of Maranhão. Data were collected from 24 dairy farms between January and December 2024 through in-person surveys administered to farmers or managers. Three systems were analyzed: traditional, medium, and high-tech, which differ in technological level, ranging from animals without dairy aptitude in traditional systems to specialized animals in more intensive systems. The farms were evaluated based on their production structure, fixed and variable costs, and economic (EOC, TOC, TC, GM, NM, P) and financial (NPV, IRR, profitability index, and profitability rate) indicators, considering a 10-year horizon and a minimum attractive rate (MAR) of 6% per year, associating productive performance with the economic return of the different systems. Only the highly technologically advanced systems were profitable, with indicators close to the MAR. Thus, the most technologically advanced dairy systems proved to be the most economically attractive, reflecting the effect of technological intensification and genetic selection on the productive performance and bioeconomic efficiency of these systems, providing support for the sustainable management of small farms in the Pre-Amazon region of Maranhão.

## Introduction

According to the Food and Agriculture Organization of the United Nations (FAO/UN), milk is the most consumed food in the world and plays a strategic role in food security and in the country’s economy. In 2023, Brazil was the sixth largest producer in the world, with 35.37 billion liters, equivalent to 4% of global production, concentrated in the states of Minas Gerais, Goiás, Paraná, Santa Catarina, and Rio Grande do Sul, which accounted for 68.63% of the total Brazilian production (EMBRAPA 2025).

In the Pre-Amazon region of Maranhão, production is still low compared to the national average, with 420,000 L in 2023, just 1.19% of the total. However, it has grown in recent decades, rising from 145,000 L in 1995 to 420,000,000 in 2023 (3.74% per year). This growth has positioned the state as the sixth largest milk producer in the Northeast (EMBRAPA 2025).

However, this growth is sustained by extensive systems with low technological standards. Cities such as Açailândia and Amarante, located within the Pre-Amazon Region of Maranhão, lead the state’s production, but with low productivity, less than 820 L/cow/year, due to the large number of cows milked. In contrast, São Francisco do Brejão, despite having a smaller herd, achieves greater efficiency, with 918 L/cow/year (IBGE 2021).

According to Silva (2023), the activity is mostly carried out on small properties (up to 100 hectares) and is essential for generating income and rural employment, but faces obstacles related to infrastructure, market, and management. Milk industrialization is still limited, informal trade prevails and reduces added value (IMESC 2023), while many farmers make decisions without financial records, confusing profit with cash flow and compromising economic sustainability.

Public policies and technical assistance have made progress, but remain insufficient. The lack of management, genetic, and nutritional technologies limits productivity gains, and pasture degradation reinforces the need for more intensive and sustainable models, especially in this region within the Legal Amazon.

In this context, the Pre-Amazon region of Maranhão stands out for concentrating a significant portion of the state’s dairy production, offering favorable conditions for expansion. However, the use of low-tech systems predominates. Given the low production efficiency and fragile economic management, it is essential to evaluate which models offer the best bioeconomic performance.

Thus, this research seeks to fill this gap by providing empirical evidence on the most sustainable systems from a technical and financial perspective. The objective of this study was to evaluate the bioeconomic efficiency of low-, medium-, and high-tech pasture-based systems in the dairy cattle production chain in the Pre-Amazon region of Maranhão.

## Materials and methods

### Location

**Fig. 1:**
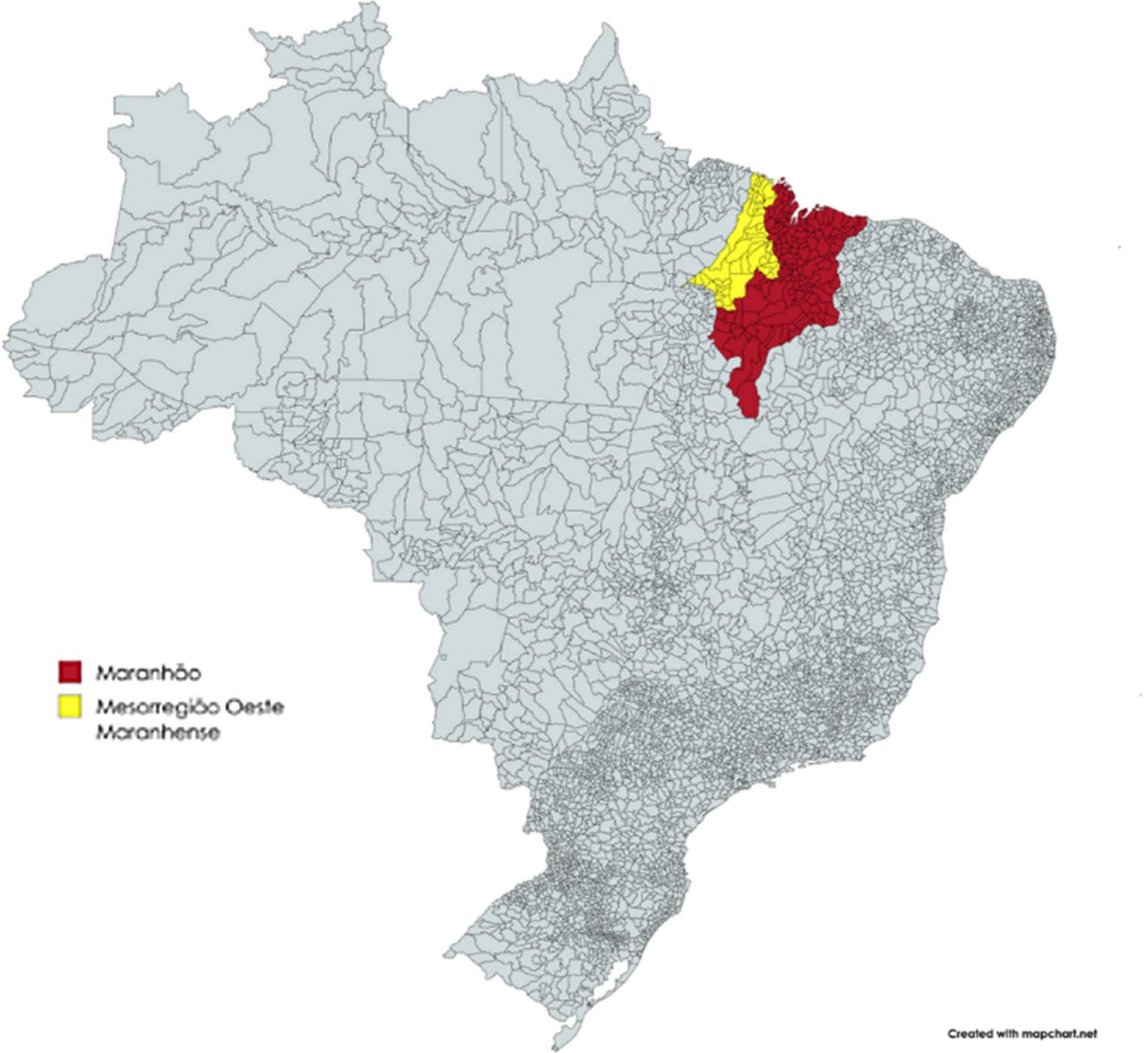
State of Maranhão with emphasis on the Pre-Amazon region

Data were collected through surveys in different pasture-based milk production systems on rural properties located in the Pre-Amazon Region of Maranhão (Fig. 1).

**Fig. 2:**
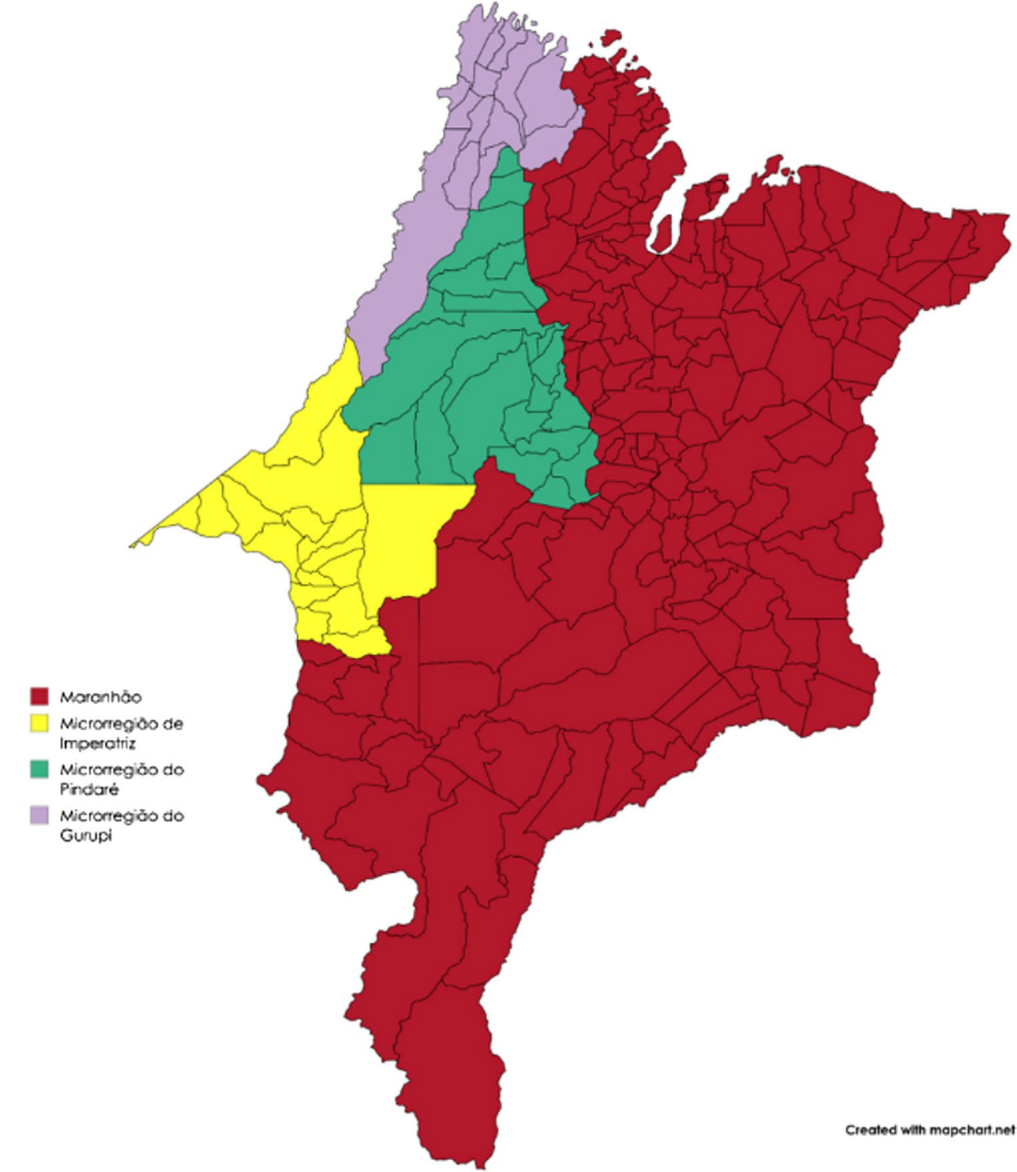
Pre-Amazon region of Maranhão, highlighting the main microregions (Imperatriz, Pindaré and Gurupi)

The Imperatriz Microregion is highlighted in yellow in Fig. 2, where this study was focused and has 16 cities, namely: Açailândia, Amarante do Maranhão, Buritirana, Cidelândia, Davinópolis, Governador Edison Lobão, Imperatriz, Itinga do Maranhão, João Lisboa, Lajeado Novo, Montes Claros, Ribamar Fiquene, São Francisco do Brejão, São Pedro da Água Branca, Senador La Roque and Vila Nova dos Martírios. The choice of the Imperatriz Microregion is justified by its relevance as a regional dairy hub and by the diversity of strategies adopted by the properties regarding handling, feeding and production management.

The information was obtained through structured surveys (ANNEX), applied in person to farmers or technical managers of the properties, from January to December 2024.

One hundred and twenty farmers from pasture-based production systems with low-, medium-, and high-lactation cows on properties of up to 70 hectares were interviewed. To deepen the economic and bioeconomic analysis, a purposive selection of 20% of the initial sample (24 farmers) was made based on two main criteria: (i) availability of complete management records, such as notes, field notebooks, and income and expense invoices; and (ii) consistency and accessibility of information related to the production system inventory.

These 24 farmers make up the “active” group, meaning those assessed with the highest degree of control and organization over their activities. These participants were chosen to ensure greater accuracy in the calculations of economic and bioeconomic indicators. Data from these properties also constitute the primary analysis of the results presented in this study.

Furthermore, detailed information was gathered on the assets used in the activity, including improvements, machinery, vehicles, equipment, and implements. For each inventory item, the acquisition value, estimated useful life, and residual value were recorded. This data is essential for measuring fixed production costs and forms the basis for calculating indicators such as depreciation, total investment, and bioeconomic efficiency.

### Characterization of production systems

Three distinct dairy production systems were considered (Table 1), defined based on the level of technification adopted by the participating properties in 2023 and 2024. The classification was established based on animal performance and productive criteria, with emphasis on the percentage of lactating cows, the average daily production per cow and the average duration of lactation.

**Table 1:** Productive characterization of the dairy production systems evaluated in 2023 and 2024

Criterion	TS	MTS	HTS
% of lactating cows	Up to 50%	Up to 70%	Up to 83%
Average production (L/cow/day)	5 L	8 L	10 L
Average days of lactation	270 days	300 days	300 days

TS – Traditional System; MTS – Medium Technification System; HTS – High Technification System

The herd composition used in this study was based on data obtained from respondents between January and December 2024, using a representative model constructed from the weighted average of the most common systems among the 120 farmers. Thus, the structure described is not identical across all properties, but it reflects the prevailing reality among the farmers in the sample.

The Traditional System had up to 50% of cows in lactation, an average of 5 L/cow/day for 270 days, kept on extensive pastures, supplemented only with mineral salt, and natural breeding without genetic selection, with a predominance of Nelore or Tabapuã bulls. The Medium-Technification System had up to 70% of cows in lactation, a production of 8 L/cow/day for 300 days, the use of managed pastures, strategic supplementation with concentrates and silage during the dry season, and natural breeding with dairy bulls such as Gir or Holstein. The High-Technification System reached up to 83% of cows in lactation, an average production of 10 L/cow/day for 300 days, with rotational pastures, fertilization and grazing control, protein-energy supplementation above 0.1% of live weight, and reproduction based primarily on FTAI using high quality bulls.

### Economic indicators

To describe the data, economic indicators were used to demonstrate the return on breeding systems, for later discussion and comparison between them, and the variables that limit the profitability of the systems. The economic data are described below, according to Silva et al. (2006), as shown in Table 2.

**Table 2:** Economic indicators used for data analysis

Indicators	Description
Total Revenue (TR)	Liter of milk produced x marketing value
Effective Operating Cost (EOC)	Variable expenses such as feed, health, animal acquisition and other costs such as electricity
Total Operating Cost (TOC)	EOC + depreciation + pro-labore (fixed costs)
Total Cost (TC)	TOC + opportunity cost (fixed cost)
Depreciation by the straight-line method	(Vi/ Vf)/n
Gross Margin (GM)	(TR – EOC)
Net Margin (NM)	(TR – TOC)
Profitability (P)	(TR – TC)
Leveling point	(TC/average animal weight/sale value)
Benefit/Cost Ratio (B/C)	(TR/TC)

Source: (Silva et al. 2006)

### Financial indicators

The financial indicators were calculated considering a 10-year horizon, applying an annual discount rate of 6%. This rate was chosen based on an estimate of the opportunity cost of capital in medium-sized agricultural activities, aligned with the minimum attractive rate (MAR). The indicators used were: Net Present Value (NPV).

$$\>NPV = - CO + \>\sum {{an} \over {(1 - MAR)}}$$

Where: C0 is the initial investment in period zero, *an* is the income stream in period *n* (in this case it is 10 years), MAR is the minimum attractive rate (6% per year). Payback was calculated based on the following formula:

$$\>Payback = {{INV} \over {\sum ({{NF1} \over {\left({1 + r} \right)1}} + {{NF2} \over {\left({1 + r} \right)2}} + \ldots \> + \>{{NFn} \over {\left({1 + r} \right)n}}}}$$

Where INV is the initial investment (R$), NF is the net cash flow in each year, *n* is the cash flow period (10 years), *r* is the discount rate (6% per year). The internal rate of return (IRR) modified internal rate of return (MIRR), profitability index (PI) and profitability rate (PR) were calculated (Motta and Caloba 2006; Frezatti 2007). The dollar conversion rate was: US$ 1.00 = R$ 6.00.

This research is quantitative, descriptive, and uses an observational approach that compares different production systems (low, medium, and high technification) according to the methodology of Marconi and Lakatos (2004). Simulations and calculations of economic and financial indicators were performed using a Microsoft Excel spreadsheet based on the model proposed by Silva et al. (2006). This tool allowed for the organization, standardization, and comparison of data across systems, respecting the production structure of each system.

## Results

The highest costs were recorded in the most technologically advanced systems, mainly due to feeding and reproduction (Table 3). In the highly technologically advanced system (83% of cows in lactation), feeding reached R$ 63,250.00 and reproduction R$ 58,000.00, values 54% and 7% higher than those of the medium technologically advanced system (70% of cows in lactation) which reached R$ 41,250.00 and R$ 54,300.00, and 289% and 16% above those with low technification (50% of cows in lactation), spending R$ 16,250.00 and R$ 50,000.00.

The higher level of technification, combined with increased feed and reproduction costs, also increased fixed costs, especially opportunity costs. In high-tech systems, this value was R$19,955.00, representing a 7% increase compared to medium-tech systems (R$18,670.00) and a 16% increase compared to low-tech systems (R$17,205.00). For the other components, health, depreciation, and maintenance, no differences were recorded, remaining the same across the three systems.

**Table 3:** Variable and fixed costs (R$) considered in dairy cattle production systems with different levels of technification

Variables	TS	MTS	HTS
Feeding (R$)	16,250.00	41,250.00	63,250.00
Reproduction (R$)	50,000.00	54,300.00	58,000.00
Health (R$)	750.00	750.00	750.00
Other Costs (R$)*	71,144.00	71,144.00	71,144.00
Depreciation (R$)	13,709.80	13,709.80	13,709.80
Maintenance (R$)	12,297.80	12,297.80	12,297.80
Opportunity Cost (R$)	17,205.00	18,670.00	19,955.00

*labor, administrative costs, electricity, fuels and lubricants, rural taxes and fees, technical assistance and consultancy services, freight and transportation. TS – Traditional System; MTS – Medium Technification System; HTS – High Technification System

The greater investment in feeding and reproduction in the more technologically advanced systems increased gross revenue by 289% compared to the more traditional systems. This increase in revenue was accompanied by an increase in variable costs, especially the effective operating cost (EOC). In systems with 83% lactating cows, EOC was 21.88% higher than in systems with 70% lactating cows and 62.4% higher than in systems with only 50% lactating cows. Total operating cost (TOC) also increased, but to a lesser extent, since the main adjustments on farms were directed to feeding and reproduction (Table 4), indicating that intensification had a greater impact on variable costs than on fixed costs.

It is important to highlight that in low-technification systems (50% lactating cows), which are commonly characterized as dual-purpose systems in tropical Latin America, revenue from the sale of calves (beef component) may represent a complementary source of income. However, in the present study, even when considering the potential contribution of calf sales, this revenue was insufficient to offset the low milk productivity and the inefficiencies associated with reduced herd specialization. This reflects a common structural limitation in traditional dual-purpose systems, where low zootechnical performance compromises overall profitability.

**Table 4:** Economic indicators in dairy cattle production systems with different levels of technification

Variables (R$)	TS	MTS	HTS
Total revenue	67,500.00	168,000.00	252,000.00
Effective Operating Cost (EOC)	88,144.00	117,444.00	143,144.00
Total Operating Cost (TOC)	114,151.60	143,451.60	169,151.60
Total cost	131,356.60	162,161.60	189,106.60
Gross margin	− 20,644.00	50,556.00	108,856.00
Net margin	− 46,651.60	24,549.00	82,849.00
Profitability	− 63,856.60	5,838.40	62,893.40
Benefit/Cost Ratio (B/C)	0.51	1.04	1.33
EOC (liter produced)	2.61	1.40	1.14
TOC (liter produced)	3.38	1.71	1.34
Total cost (liter produced)	3.89	1.93	1.50

TS – Traditional System; MTS – Medium Technification System; HTS – High Technification System

In low-tech systems, all margins (gross, net, and profitability) were negative. When the percentage of lactating cows was increased to 70%, (mid-tech systems), a 40% increase compared to the more traditional system, margins became positive, indicating greater economic viability. With the increase to 83% lactating cows, 66% above the 50% system, the activity became even more attractive, reaching a profitability of up to R$62,893.40.

The benefit-cost ratio also showed the same behavior, where properties with 50% lactating cows had a ratio lower than 1, that is, for each real invested, the return obtained was lower than the amount invested. Unlike systems with 70% and 83% lactating cows, when it was found that for each real invested, a return of 0.04 and 0.33 cents was observed, respectively (Table 4).

The greater efficiency in diluting production costs in the more technologically advanced systems (83% lactating cows) resulted in lower costs per kg of milk produced, requiring only R$1.50/kg to cover all costs. In contrast, in less intensive systems, with 50% lactating cows, the cost per kg of milk to cover all costs and obtain a profit reaches R$3.89/kg, highlighting the economic advantage of intensification.

When carrying out long-term financial analyses considering the net present value (NPV), it was observed that, in systems with up to 70% of lactating cows, investments were not covered in the 10-year period, presenting all negative financial indicators (Table 5).

Although dual-purpose systems may partially benefit from the commercialization of male calves or surplus heifers, the financial projections demonstrate that such additional income does not substantially modify long-term investment feasibility when milk productivity remains low. This reinforces the notion that improving herd structure and increasing the proportion of lactating cows are more decisive factors for economic sustainability than relying on secondary beef revenue.

**Table 5:** Financial indicators in dairy cattle production systems with different levels of technification

Variables	TS	MTS	HTS
NPV - Net present value (R$)	− 597,968.13	− 84,712.46	334,922.90
Discounted Payback (years)	10.00	10.00	2.00
IRR - Internal rate of return (% per year)	0.00	0.00	7.00
Profitability index	0.00	0.00	6.00
Rate of return (%)	0.00	0.00	3.00

TS – Traditional System; MTS – Medium Technification System; HTS – High Technification System

On the other hand, when livestock performance increases, reaching 83% of lactating cows in relation to the total herd, investments become viable over a 10-year horizon (NPV > 0). In these systems, the investment would be recovered in only 2 years, as indicated by Payback. Furthermore, the profitability rate was 3%, and the internal rate of return reached 6% per year, configuring the most favorable scenario among those evaluated (Table 5).

The system that responded most positively to the sensitivity analysis, considering the appreciation and depreciation of the price of a liter of milk of up to 20%, was the most technologically advanced system with 83 lactating cows in the total herd (Table 6). Meanwhile, the low-technification system did not present positive results in any scenario.

**Table 6:** Sensitivity analysis of dairy cattle production systems with different levels of technification

Variables	10	20	30	-10	-20	-30
TS						
NPV - Net present value (R$)	− 421,657.25	− 371,976.66	− 322,296.07	− 521,018.42	− 570,699.01	− 620,379.60
NPVa (R$)	− 57,289.71	− 50,539.71	− 43,789.71	− 70,789.71	− 77,539.71	− 84,289.71
Payback (years)	10	10	10	10	10	10
IRR - Internal rate of return (% per year)	0	0	0	0	0	0
Profitability index	0	0	0	0	0	0
Rate of return (%)	0	0	0	0	0	0
MTS						
NPV - Net present value (R$)	207,998.20	331,647.66	455,297.12	− 39,300.73	− 162,950.19	− 286,599.65
NPVa - (R$)	28,260.29	45,060.29	61,860.29	− 5,339.71	− 22,139.71	− 38,939.71
Discounted Payback (years)	3.16	2.25	1.75	10	10	10
IRR - Internal rate of return (% per year)	33.72	47.82	61.41	-1.08	0	0
Profitability index	2.63	3.59	4.56	0.69	-0.27	-1.24
Rate of return (%)	162.53	259.14	355.76	-30.71	-127.33	-223.94
HTS						
NPV - Net Present Value (R$)	726,148.33	911,622.52	1,097,096.71	355,199.94	169,725.75	− 15,748.45
VPLa - (R$)	98,660.29	123,860.29	149,060.29	48,260.29	23,060.29	− 2,139.71
Discounted Payback (years)	1.20	0.96	0.50	2.14	3.62	10
IRR - Internal rate of return (% per year)	90.53	110.30	130.03	50.43	29.16	3.32
Profitability index	6.67	8.12	9.57	3.78	2.33	0.88
Rate of return (%)	567.40	712.33	857.25	277.55	132.62	0

TS – Traditional System; MTS – Medium Technification System; HTS – High Technification System

## Discussion

Feeding has been identified by farmers as the main weakness of dairy farming, especially due to its direct impact on production costs (Silva 2013). This aspect was confirmed in the present study, in which more technologically advanced systems increased the level of supplementation, such as the use of feed, increasing variable costs (EOC), but ensuring a greater number of lactating cows (70 and 83% of lactating cows) and, consequently, better production efficiency, both in productivity and profitability, by better meeting the nutritional requirements of the category.

Among the less technologically advanced systems (50% of cows in lactation), it was observed that feeding is based almost exclusively on non-irrigated and unfertilized pastures, used year-round as the sole forage resource, associated with only mineral supplementation, sometimes not specific to the category, which limits production efficiency and sustainability. Thus, although representing one of the greatest economic challenges for farmers, adequate feeding is also the main lever for intensification and viability of dairy systems.

Another significant production cost across systems was reproduction, which, despite entailing additional costs, represents an essential strategic component for the economic sustainability of the systems. In less technologically advanced systems, low reproductive efficiency limits the proportion of lactating cows (50% of lactating cows) and reduces production potential, compromising farm profitability. In these cases, increasing the number of animals, a traditional practice in the region (IBGE 2021), does not guarantee a greater financial return, because without improving the herd’s genetic potential and adequate reproductive management, individual cow productivity remains low (Silva et al. 2012).

In contrast, in medium- and high-tech systems, the adoption of structured reproductive strategies, such as artificial insemination and systematic management, allows for greater calving regularity and an increase in the proportion of lactating cows. These procedures, combined with genetic improvement using Gir or Holstein cattle, ensure higher productivity per cow and, consequently, an effective financial return, demonstrating that herd expansion only becomes economically advantageous when correlated with productive efficiency (Kompas and Che 2006).

According to the Maranhão dairy industry assessment, milk production growth continues predominantly through herd expansion, maintaining extensive characteristics (IMESC 2023). This scenario reinforces the need for a transition to more intensive systems, with breeding practices, strategic nutritional management, preventative health, and efficient pasture utilization. Intensification, although it implies increased feed and reproduction costs, is crucial to increasing production efficiency, making farms economically viable, and fostering regional competitiveness against states with higher technological levels, such as Minas Gerais, Goiás, and Paraná.

The results of this study corroborate the report by the Maranhão Institute of Sociological and Cartographic Studies (IMESC) (2023) regarding the increase in milk productivity in Brazil, even with the reduction in herd size. In systems with a higher proportion of lactating cows relative to the total herd (70 to 83%), improvements in production efficiency were observed, reflecting the behavior observed nationally linked to the increase in productivity of milked cows. These systems adequately cover production costs and present positive margins, making the activity economically attractive in the short, medium, and long term, in addition to allowing both the maintenance and expansion of investments, highlighting the strategic role of intensification in the viability of dairy systems.

In contrast, in less technologically advanced systems, with only 50% of cows in lactation, performance followed the pattern still prevalent in Maranhão, where milk production is primarily influenced by herd growth rather than individual productivity increases. Despite lower costs in these systems, low production efficiency compromises economic viability, generating negative margins and the risk of abandoning the activity. This situation directly impacts family income, reduces rural job creation, and diminishes the sector’s competitiveness in the state. Furthermore, limited technical assistance, low managerial training, and restricted access to rural credit (Bezerra et al. 2017) negatively impact the modernization of dairy farming in Maranhão, reinforcing the need for policies and strategies that promote greater efficiency and sustainability in the sector.

In tropical Latin American contexts, low-technification dairy farms are frequently structured as dual-purpose systems, where milk and beef production coexist as complementary income sources. In such systems, the commercialization of male calves and surplus heifers may represent an additional revenue stream, partially compensating for lower milk productivity. However, although calf sales can contribute to cash flow, this income component alone is generally insufficient to offset structural inefficiencies associated with low proportions of lactating cows, limited reproductive control, and reduced nutritional management.

Traditional dual-purpose systems often prioritize herd size over productive efficiency, which limits milk yield per animal and reduces the capacity to dilute fixed costs. Consequently, even when accounting for the beef component, overall profitability remains constrained when biological efficiency is low. These findings suggest that improving herd structure and increasing the proportion of lactating cows are more decisive for long-term economic sustainability than relying on secondary beef revenue.

Due to structural, technical, and logistical limitations that affect production flow, IMESC (2023) found, during visits to establishments in the Imperatriz Microregion, that milk production often has a cost-benefit ratio of less than 1, meaning that return does not cover production costs. This situation was confirmed in the present study in the less technologically advanced systems, with a cost-benefit ratio of 0.51, indicating low economic return and a risk of non-viability. In contrast, in the more technologically advanced systems, with 70 to 83% of cows in lactation, the cost-benefit ratio exceeded R$1.00, demonstrating greater economic attractiveness. These results provide a basis for analyzing financial indicators such as Net Present Value (NPV) and Internal Rate of Return (IRR), which allow us to assess the viability and profitability of investments at different levels of technological development.

Only in the highly technological system (83% of cows in lactation) was the Minimum Attractiveness Rate (MAR) of 6% higher than the IRR of 6%, according to Kreuz et al. (2008). This shows that expectations are that there will be more gain in investing in the project than leaving the money invested, for example, in savings accounts yielding 6% per year (MAR). Furthermore, Payback was shorter than the maximum recovery period for the activity (10 years) in this system, favoring the acceptance of projects. According to Barbieri et al. (2016), the *Return pay on equity* is not a measure of profitability; it only measures the time required for capital recovery. Therefore, it must be analyzed alongside other indicators such as NPV and IRR.

In a scenario of investments and financial analysis tech systems (83% lactating cows) were more attractive than those with medium-tech systems (70% lactating cows). This is because both the NPV and IRR found in higher -tech systems were positive, revealing a superior capacity to generate value on invested capital and robustness against fluctuations in the production scenario. The intensification of dairy farming implies an increase in variable costs, but provides greater dilution of fixed costs and a faster return on invested capital, provided productivity keeps pace with this growth. This scenario favors systems with a greater proportion of lactating cows relative to the total number of cows, which, by requiring greater investment in nutrition and management, also maximizes the system’s long-term profitability (Nunes et al. 2024).

A sensitivity analysis of the milk production systems revealed significant differences in economic resilience to milk price fluctuations. In the most technologically advanced systems, it was found that, even with a reduction of up to 20% in milk price, the project remained economically viable. This result is corroborated by studies indicating that systems with higher technological levels and better reproductive management have a greater capacity to adapt to market fluctuations, maintaining profitability even in adverse scenarios (Peres et al. 2009).

On the other hand, in more traditional systems, a mere 10% drop in milk prices compromised the project’s economic viability, highlighting the vulnerability of these systems to market fluctuations. This sensitivity is compounded by low productivity, high fixed costs, and a lack of risk mitigation strategies, characteristics common in less intensified systems (Lopes 2023).

The findings reinforce that the intensification of production, through the adoption of appropriate technologies, efficient management and reproductive control, not only increases productivity, but also provides greater economic stability to milk production systems.

Hi-tech production systems with up to 83 lactating cows present greater economic and financial attractiveness, with higher profit and NPV values than traditional and medium-tech systems. This demonstrates that intensification with feeding, reproduction, and supplementation constitute viable strategies for increasing the bioeconomic efficiency of dairy cattle farming on small farms in the Pre-Amazon region of Maranhão. While maximizing return per hectare, the system’s success depends on proper management, which mitigates risks related to fluctuations in milk sales prices, given the high production costs.

## Data Availability

The datasets generated and/or analyzed during this study are not publicly available due to the non-finalization of reviewers’ considerations and the non-submission of the thesis to the graduate program, but can be obtained from the corresponding author upon reasonable request.
